# COVID-19-Associated Coagulopathy: A Case Report of Thrombosis despite Therapeutic Anticoagulation

**DOI:** 10.1155/2020/8876932

**Published:** 2020-10-17

**Authors:** Habiba Hussain, Matthew Sehring, Bhagat Singh Aulakh

**Affiliations:** ^1^Department of Internal Medicine, University of Illinois College of Medicine at Peoria, Peoria, IL, USA; ^2^Department of Pulmonary & Critical Care Medicine, University of Illinois College of Medicine at Peoria, Peoria, IL, USA

## Abstract

The Coronavirus disease (COVID-19) pandemic, caused by Severe Acute Respiratory Syndrome Coronavirus 2 (SARS-CoV-2), has led to tremendous morbidity and mortality. Various inflammatory markers have been monitored and considered to be associated with disease prognosis. One of the major sources of comorbidity involved has been development of thrombosis alongside the infection. This prothrombotic phenomenon considered, COVID-19-associated coagulopathy (CAC), has been the center of discussion in dealing with this infection. There still remains ambiguity regarding management guidelines for thromboprophylaxis dosing and therapeutic anticoagulation. We present a case of severe SARS-CoV-2 infection complicated by thrombosis despite therapeutic anticoagulation contributing to prolonged intensive care unit and hospital stay.

## 1. Introduction

The COVID-19 pandemic has led to tremendous morbidity and mortality. SARS-CoV-2 is a positive sense single-stranded RNA virus genome with an envelope surrounded by surface glycoprotein, spike or S protein, responsible for tropism towards angiotensin-converting enzyme 2 (ACE2) receptor of human respiratory epithelium [1]. Increasing evidence of postmortem findings for microvascular and thromboembolic phenomenon seen in patients with COVID-19 prompted the discussion for the role and extent of venous thromboembolic (VTE) prophylaxis [1, 2].

## 2. Case Description

**Figure 1:**
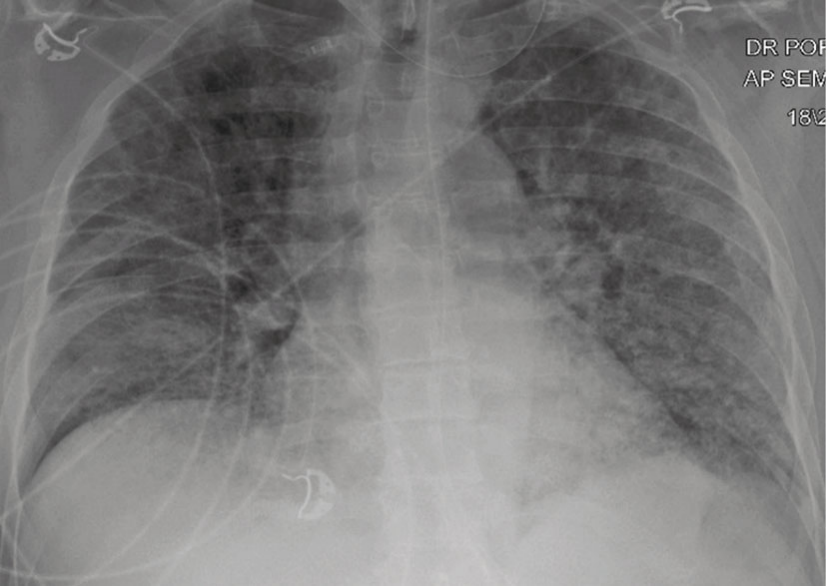
Initial bilateral pulmonary opacities.

**Figure 2:**
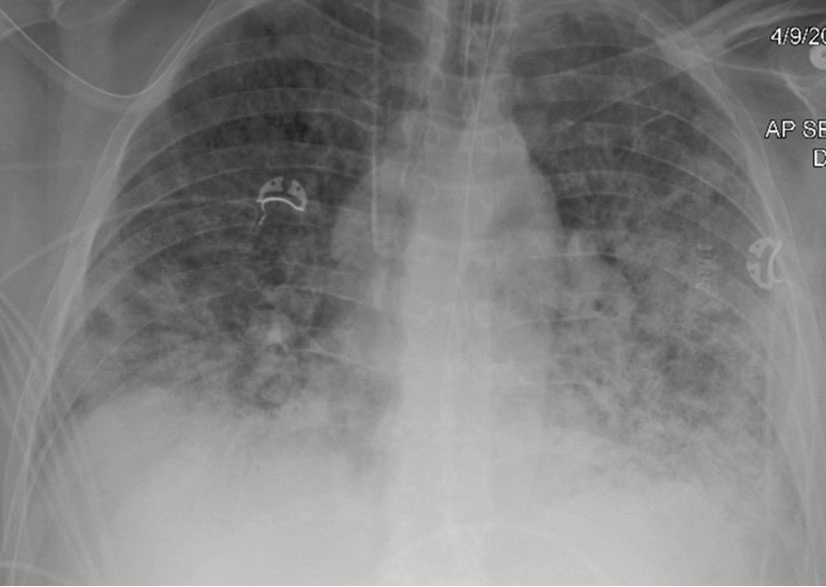
Worsening bilateral diffuse opacities.

A 59-year-old male with a past medical history of 30-pack per year tobacco abuse and a recent history of vaping presented with fever, cough, shortness of breath, and generalized fatigue. He was found in acute hypoxic respiratory failure requiring mechanical ventilation (pressure-regulated volume control (PRVC) mode, respiratory rate (RR) 16/minute, tidal volume (TV) 480 mL, positive end expiratory pressure (PEEP) 7 centimeters of water, fractional inspiratory oxygen (FiO_2_) 80%, and partial arterial oxygen (PaO_2_/FiO_2_) < 100). He was further noted to be in septic shock likely secondary to pneumonia as chest X-ray (CXR) showed bilateral pulmonary opacities (Figure 1). He received empiric therapy for community-acquired pneumonia with ceftriaxone and azithromycin. Repeat CXR upon worsening symptoms showed increased bilateral diffuse opacities concerning for acute respiratory distress syndrome (ARDS) (Figure 2). During his acute decompensation, testing for SARS-CoV-2 resulted positive. In an effort to optimize respiratory status with available modalities at the time he received diuresis, hydroxychloroquine and trial of prone positioning.

**Figure 3:**
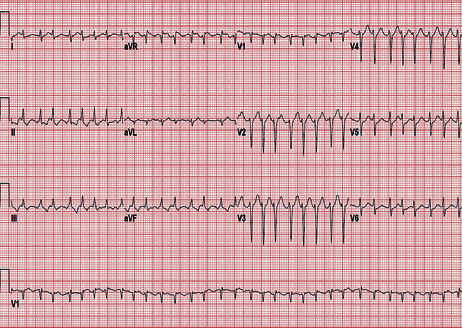
Supraventricular tachycardia with ventricular rate > 190 bpm.

**Figure 4:**
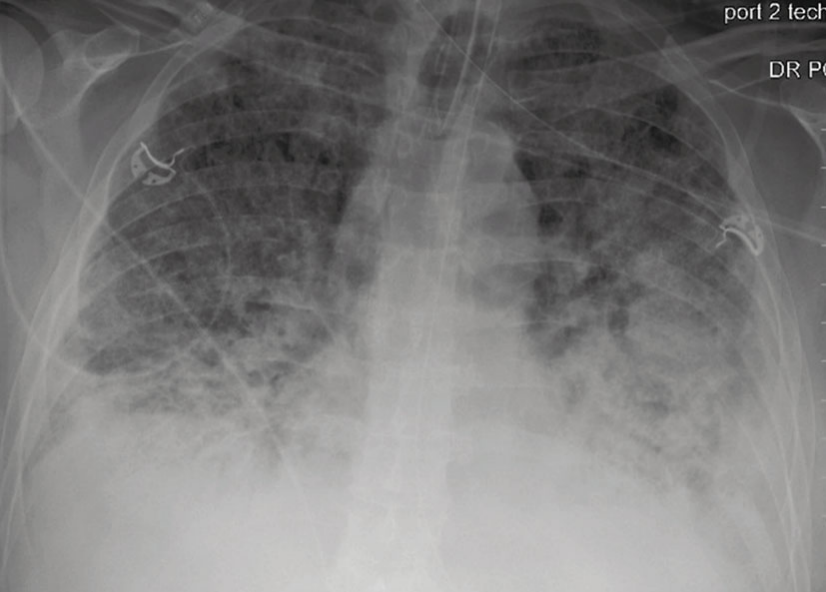
Diffuse bilateral airspace opacities with dense opacification in the left midlung.

**Figure 5:**
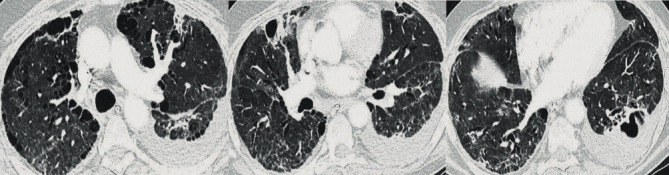
Bilateral lower lobe traction bronchiectasis, evidence of emphysema, post ARDS fibrosis with interlobar septal thickening from recent viral infection, diffuse subpleural cysts, multiple thick-walled cavitary lesions concerning for bacterial superinfection with intrapulmonary abscesses in the lower lobes.

**Figure 6:**
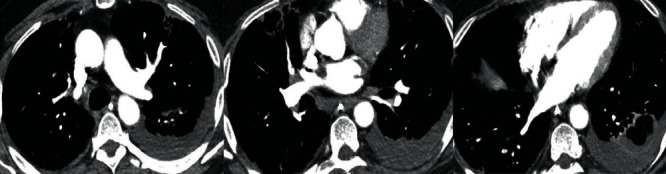
Filling defects in the right lower lobe, right middle lobe, and left upper lobe subsegmental arteries.

Meanwhile, in light of hypercoagulable pattern observed in SARS-CoV-2 infections, he was maintained on adequate VTE prophylaxis with enoxaparin 40 mg daily (sequential organ failure assessment: SOFA score 5, sepsis-induced coagulopathy: SIC score 2) [1, 2]. During the course of intensive care unit (ICU) stay, he occasionally developed leukocytosis (peak 24,000 with neutrophilic predominance), C-reactive protein (CRP) peak at 37.4 mg/dL, and ferritin peak at 589 ng/mL. The most remarkable feature noted was an elevated D‐dimer > 20 mcg/mL on the day of presentation, declining to 8.76 mcg/mL the next day and thereafter improved to plateau around 3-5 mcg/mL. Due to failure of improvement, requiring increased vasopressor and ventilatory support, he received empiric treatment for superimposed ventilator-associated pneumonia and immunomodulation with the interleukin 6 (IL6) inhibitor tocilizumab which showed transient clinical improvement. He again worsened to developed unstable supraventricular tachycardia (SVT) requiring electrical cardioversion for return to sinus rhythm (Figure 3). With the knowledge of elevated D-dimer, worsening oxygenation, and new unstable arrhythmia indicative of microvascular thrombosis depicting end-organ dysfunction, he was empirically started on therapeutic dosing of enoxaparin maintaining target anti-Xa level (enoxaparin increased from 40 mg daily to 90 mg twice daily on day 14 of admission, while the d-Dimer was 5.68 mcg/mL). His mechanical ventilatory support requirements continued to rise (higher PEEP 18-20 cm water and FiO_2_ 80-90%) despite treatment and therapeutic anticoagulation; hence, he was started on convalescent plasma and high-dose steroids on day 27 of admission (Figure 4). Following a long course of critical illness, he was able to be weaned off of vasopressors and ventilatory support. Eventually, he was tested and found negative for COVID-19 for the first time on day 33 of admission, with a repeat negative test three days later. In an effort to transition off therapeutic anticoagulation dosing after being tested negative, he underwent computed tomography with angiography (CTA) which showed bilateral subsegmental pulmonary emboli, lower lobe traction bronchiectasis, fibrotic changes, and diffuse subpleural cysts. He was also noted to have multiple thick-walled cavitary lesions in the right middle lobe, bilateral bases, and left basilar cavitary abscess 5.4 × 3.5 cm (Figures 5 and 6). With the evidence of new bilateral segmental pulmonary emboli, he was transitioned from therapeutic enoxaparin to apixaban 5 mg twice daily for around 6 months until further evaluation.

## 3. Discussion

Implication of SARS-CoV-2 infection with thromboembolism or disseminated intravascular coagulation (DIC) has been extrapolated from sepsis-induced coagulopathy (SIC). The cause is thought to be triggered by a host inflammatory reaction leading to consumptive coagulopathy, since the virus itself is not known to have a procoagulant effect [1]. Preliminary data emerging from Wuhan, China, and Netherlands have demonstrated evidence of coagulopathy with abnormal parameters such as prothrombin time, activated prothrombin time, D-dimer, fibrinogen, ESR, CRP, thrombocytopenia, and IL6 levels [1–7]. This propensity of abnormal values is seen more predominantly in ICU patients dealing with severe SARS-CoV-2 infection (respiratory rate > 30/minute, saturation < 93% on room air, and partial pressure of arterial oxygen/fraction of inspired oxygen PaO_2_/FiO_2_ < 300 mmHg) [1]. The International Society on Thrombosis and Haemostasis (ISTH) devised SIC score for diagnosis and evaluation for risk of transition to DIC [1]. Elevated levels of D-dimer have been associated with increased mortality risk [2, 8–10]. Thromboprophylactic heparin used in patients with severe COVID-19 showed decreased mortality in patients with SIC score > 4 and patients with D‐dimer > 3 mcg/mL likely by mitigating the risk of microvascular thrombosis or pulmonary embolism. However, it is imperative to note that underlying cytokine storm in addition to coagulation activation also contributes to elevated levels of D-dimer, as against solely a thromboembolic phenomenon [1]. Our case and reports from data available from Netherlands and France suggest higher propensity of pulmonary embolism in the intensive care unit with SARS-CoV-2 infection while patients received standard dose of VTE prophylaxis [1, 11]. This has prompted a shift in practice for increase to intermediate dosing VTE prophylaxis with enoxaparin (0.5 mg/kg twice daily) or heparin (7500 U three times daily) [1, 11–13].

SIC score has been based on the effect of inflammatory storm which is expected to resolve with the treatment of sepsis, therefore indicating likely benefit with transient prophylaxis. However, SARS-CoV-2 infection in the setting of absence of definitive treatment has a more prolonged course hence longer duration of anticipated inflammatory storm. With reported cases of thrombosis despite receiving VTE prophylaxis and continued challenge in the availability of appropriate imaging in SARS-CoV-2 infection, it raises concern for the need of empiric therapeutic anticoagulation in select cases. This is in effort to overcome the development of microvascular thrombosis and end-organ dysfunction [1, 2]. Therapeutic anticoagulation is currently only considered in the setting of new worsening respiratory status with evidence of right heart strain or thrombosis on extremity duplex.

We present the case of a patient with severe SARS-CoV-2 infection requiring around 25 days of ventilatory support who had presented with significantly elevated level of D‐dimer > 20 mcg/mL. He was maintained on VTE prophylaxis since initial presentation and was switched to empiric therapeutic anticoagulation (day 14 of admission) upon development of worsened respiratory decline and new unstable arrhythmia. He remained on enoxaparin 90 mg twice daily for the majority of ICU stay. Despite receiving therapeutic anticoagulation, he was noted to develop bilateral pulmonary emboli likely contributing to protracted ICU course. This presentation raises concerns regarding severity of cytokine storm as evidenced from the marked elevation of D-dimer, fibrinogen, CRP, and ferritin levels and its effect on COVID-19-associated coagulopathy. This also questions the benefit of VTE prophylactic dosing in preventing microvascular thrombosis or end-organ dysfunction in severe SARS-CoV-2 infection where therapeutic anticoagulation may also be unable to prevent thrombosis.
